# Effectiveness of Custom-Designed 3D-Printed Drill Guides in the Treatment of Lateral Humeral Condylar Fractures in a French Bulldog Bone Model

**DOI:** 10.3390/vetsci12090888

**Published:** 2025-09-14

**Authors:** Jirawat Srikusalanukul, Nattapon Chantarapanich, Chaiyakorn Thitiyanaporn

**Affiliations:** 1 Kasetsart University Veterinary Teaching Hospital Bangkhen Campus, Faculty of Veterinary Medicine, Kasetsart University, Bangkok 10900, Thailand; jirawat.sriku@ku.th; 2 Department of Mechanical Engineering, Faculty of Engineering at Sriracha, Kasetsart University, Sriracha Campus, Chonburi 20230, Thailand; nattapon@eng.src.ku.ac.th; 3 Department of Companion Animal Clinical Science, Faculty of Veterinary Medicine, Kasetsart University, Bangkok 10900, Thailand

**Keywords:** humeral condylar fracture, French bulldogs, three-dimensional printing, drill guide

## Abstract

The French Bulldog is a breed predisposed to lateral humeral intercondylar fractures. A transcondylar lag screw combined with an epicondylar pin is most commonly used for their repair. However, improper screw and pin placement is common. This study used a 3D-printed plastic bone model, designed from a 5-year-old 19.5 kg French Bulldog’s humerus. Then, a 3D-printed drill guide was designed specifically for a lateral humeral condylar fracture. The bone models were divided into two groups: the first group included the use of the 3D-printed drill guide during the screw and pin placement, and the second group did not. After implantation, we compared the angle deviation of the transcondylar screw and the epicondylar pin, as well as the exit point translation of the transcondylar screw. The results showed differences between the two groups. The first group exhibited a lower angle deviation than the second group. Additionally, the exit point translation of the transcondylar screw was reduced in the drill guide group. The use of a custom-designed 3D-printed drill guide significantly improved the accuracy of the transcondylar screw and epicondylar pin placement. This advancement could reduce the incidence of postoperative complications.

## Simple Summary

Three-dimensional printing technology is increasingly utilized to improve surgical accuracy, including in orthopedic surgeries. However, no 3D-printed surgical guides are available for repairing lateral humeral condyle fractures in French Bulldogs or other breeds. Our experiment designed a 3D-printed drill guide for this purpose, and the results showed that using the drill guide improved accuracy. In the future, when animals have lateral humeral condyle fractures, our designed 3D-printed drill guide can be used and adjusted for reduced surgical time.

## 1. Introduction

Humeral fractures constitute 8–12% of all fractures in dogs, with approximately 40% occurring in the condylar region [1], categorized as lateral condyle fractures (34–67%) [2], medial condyle fractures (6.9–11%), and intercondylar fractures (25.9–35%) [1]. In previous studies, Spaniel breeds have been identified as having a higher risk of humeral intracondylar fissures due to incomplete ossification of the humeral condyle, and recent studies indicate that French Bulldogs are also increasingly affected by this condition, resulting in a higher incidence of humeral condylar fractures [3,4].

Several stabilization methods are utilized for humeral condylar fractures, including transcondylar screws inserted in a lag fashion [5,6], pins [7], wire fixation [8], Rush pins, various applications of K-wires, self-compressing Orthofix pins [9], cannulated screws [10], closed reduction with subsequent internal fixation [11], conventional plates [12], and AdhFix plates [13]. Among these methods, transcondylar screw fixation remains the most used technique. Given the high complication rates associated with these fractures, particularly the persistence of fracture gaps after fixation, the accurate placement of lag screws is crucial. Recent advancements aimed at improving the screw placement accuracy include the use of aiming guides, fluoroscopic guidance [14], and customized 3D-printed surgical guides [15].

The integration of three-dimensional (3D) technology into surgical procedures requires the acquisition of imaging data such as CT scans or MRIs, which are saved in Digital Imaging and Communications in Medicine (DICOM) format. These files are imported into Computer-Aided Design (CAD) software to reconstruct bone models and design customized surgical guides [16], which are subsequently produced using 3D printing technology. The application of 3D technology is increasing, providing advantages in surgical education, preoperative planning, and intraoperative execution [17]. It enhances the surgical accuracy [18], reduces the risk of iatrogenic injuries, and shortens the operative times.

No studies have specifically investigated the application of 3D-printed drill guides for the treatment of lateral humeral condylar fractures. Therefore, the present study was undertaken with the objective of designing a 3D-printed drill guide for this type of fracture. The hypothesis of this study is that the use of a 3D-printed drill guide will result in more accurate placement of transcondylar screws and epicondylar pins, compared to conventional freehand techniques, as measured by the deviation angles of the transcondylar screw and epicondylar pin.

## 2. Materials and Methods

### 2.1. Humeral Model Preparation

**Figure 1:**
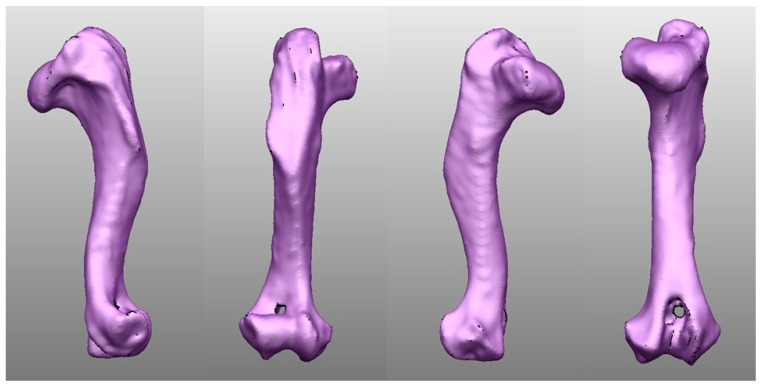
The right humerus of the French Bulldog used in this study is shown as an STL file within 3D Computer-Aided Design (CAD) software. This figure shows the lateral, caudal, medial, and cranial sides of the right humerus from left to right.

The right humerus model in this study was derived from a French Bulldog, aged 5 years and 9 months, weighing 19.5 kg, who had been presented to the Kasetsart University Veterinary Teaching Hospital Bangkhen campus. The dog underwent a CT scan for non-orthopedic-related medical issues, and the data were stored in the Digital Imaging and Communications in Medicine (DICOM) [19] format. During the scan, the dog was positioned in sternal recumbency with its limb extended in a natural straight alignment. The CT scans were acquired using a slice thickness of 0.625 mm. The DICOM files were imported into an open-source image analysis software platform (3D Slicer, version 5.2.2; The Slicer Community, MA, USA), and the soft tissues and other skeletal structures were removed to isolate the humerus. The segmented humerus model was subsequently exported and saved as a stereolithography (STL) file. The STL file was imported into 3D Computer-Aided Design (CAD) software (PowerShape, version 2023; Autodesk, San Rafael, CA, USA) (Figure 1), which was used to cut the humerus to create a lateral humeral condyle fracture. Subsequently, we imported an STL file of the humerus with the lateral condyle fracture to 3D printer software (Bambu studio; Bambu Lab, Shenzhen, China) associated with a 3D printer (Bamboo X1 0.4 nozzle printer; Bambu Lab, China), and the humerus was printed using polylactic acid (PLA) material to resemble a real bone, consisting of the bone cortex and the medullary canal, with the bone cortex thickness at 2 mm. During the printing process, the layer height was set to 0.2 mm with a sparse infill grid pattern at 15% density. The printing speeds were set as follows: first speed at 50 mm/s, first layer infill at 105 mm/s, outer wall at 200 mm/s, inner wall at 300 mm/s, and sparse infill at 270 mm/s. After the bone model was printed, the support was removed using cutting pliers.

### 2.2. Three-Dimensional-Printed Patient-Specific Drill Guide Creation

**Figure 2:**
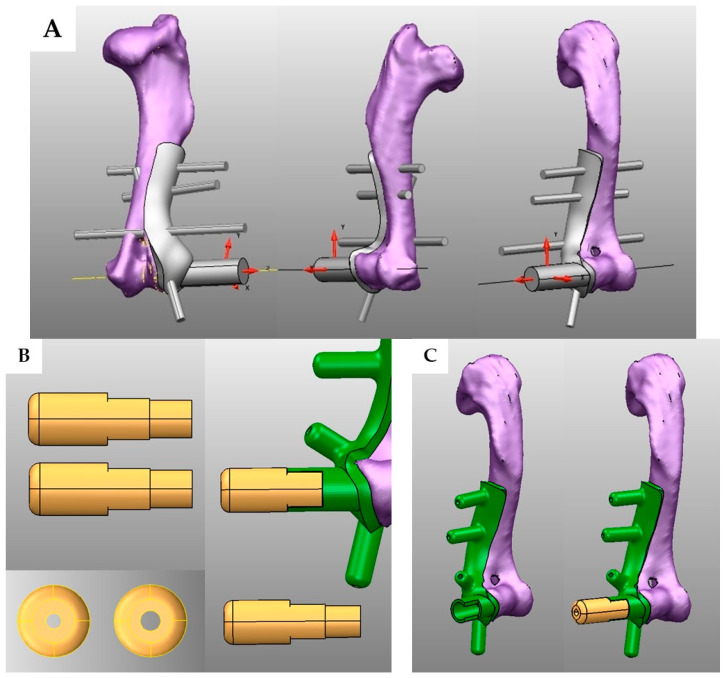
This is an example of the process of designing a 3D-printed drill guide using Computer-Aided Design (CAD) software. The design of the three-dimensional printed drill guide is shown in various views. The guide, represented in gray, was designed in a cylindrical shape. The larger cylinder corresponds to the insertion site for a 2.7 mm screw, while the remaining smaller cylindrical components are intended for the placement of 2.0 mm K-wires (**A**). The yellow color of the drill guide is the drill hole with a size of 2.7 mm and the gliding hole with a size of 2.0 mm. The design is the same for both, with hole size as the only difference (**B**). The green area represents the 3D-printed drill guide, which is attached to the humerus. The yellow portion indicates an additional component used for guiding the drilling process at the humeral condylar region. This guiding component includes two sizes of drill sleeves: 2.0 mm and 2.7 mm (**C**).

The STL file of the humeral bone model was imported into 3D Computer-Aided Design (CAD) software. A patient-specific drill guide was designed to close the surface of the lateral humeral condyle fracture and the adjacent humeral bone to ensure a secure and accurate fit. Critical anatomical landmarks were identified, with the entry point for the transcondylar screw positioned at the midpoint between the lateral epicondyle and the articular surface and the exit point located at the midpoint between the medial epicondyle and the articular surface [20]. For epicondylar pin placement, a pin trajectory was created from the distal aspect of the lateral epicondyle, directed caudomedially across the fracture line, with the tip of the pin terminating within the medial aspect of the humeral medullary canal (Figure 2A).

The main point of the drill guide was designed with two functional drilling holes: a 2.7 mm gliding hole for the insertion of the transcondylar screw and a 2.0 mm hole for drilling through the medial condyle to create a lag effect. It was designed as a two-piece device that can be easily changed (Figure 2B). Additionally, a 2.0 mm hole was incorporated into the lateral epicondyle for the insertion of a K-wire. The minor point was designed as a K-wire hole of 2.0 mm to secure the drill guide. To reduce the fractured bone fragments’ screw and pin placement, three K-wire holes were included: two on the humeral shaft and one on the fracture fragment. These fixation holes were carefully positioned to ensure that the K-wires would not be parallel to each other, thereby increasing the stability of the drill guide during use (Figure 2C). Subsequently, we imported an STL file of the 3D-printed drill guide to 3D printer software and printed it using PLA material.

### 2.3. Implantation Method

**Figure 3:**
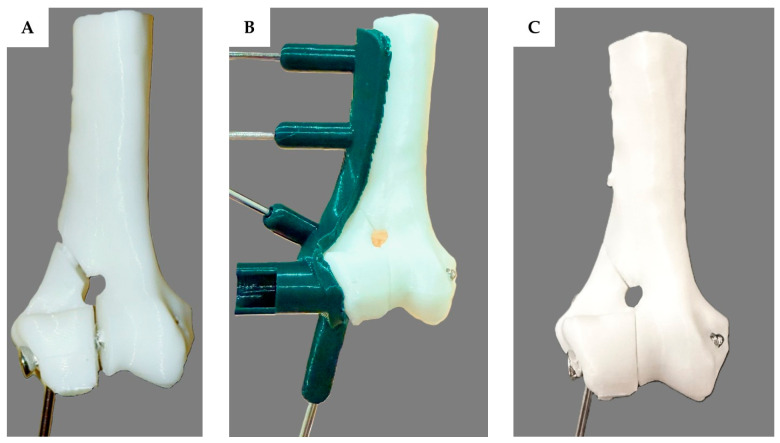
A plastic bone model with a transcondylar screw and epicondylar placement to repair a lateral humeral condyle fracture. When the transcondylar screw is inserted without using the 3D-printed drill guide, an intercondylar fracture gap can be observed (**A**). However, when the transcondylar screw is inserted using the 3D-printed drill guide (**B**), which is green in color, no intercondylar fracture gap is observed. After the implant was placed, the 3D-printed drill guide was removed (**C**). There was no fracture gap in the intercondylar area and the lateral supracondylar crest.

The testing was divided into two groups, with each group consisting of 30 humeral bone models. The implant was inserted into all 60 humeral bone models by an orthopedic surgeon. For the first group, the surgeon inserted the transcondylar screw and placed the epicondylar pin without using the 3D-printed drill guide. The humerus and fracture fragments were manually reduced and stabilized using bone holding forceps. Drilling was performed freehand, beginning with a 2.0 mm drill bit to penetrate both the lateral and medial epicondyles, followed by enlargement of the lateral epicondyle hole with a 2.7 mm drill bit. A 2.7 mm transcondylar screw was inserted, and an epicondylar pin was placed using a 2.0 mm K-wire as the final step (Figure 3A). For the second group, the surgeon inserted the transcondylar screw and epicondylar pin using the 3D-printed drill guide. In this group, the fracture fragment was reduced and aligned with the drill guide using bone reduction forceps and subsequently secured to the fracture fragment with the guide by 2.0 mm K-wire. The main humerus bone was then assembled and closely attached to the guide, followed by the placement of two additional 2.0 mm K-wires at the designated fixation points. Drilling was then performed through the lateral condylar region using a 2.0 mm drill bit following the 2.0 mm drill guide to create a tunnel through both the lateral and medial epicondyles. The drill bit and drill guide were subsequently switched to a 2.7 mm size, and drilling continued through the lateral epicondyle. The depth of the hole was measured with a depth gauge. A 2.7 mm transcondylar screw was inserted, and finally, an epicondylar pin was placed through the guide tunnel using a 2.0 mm K-wire (Figure 3B,C).

### 2.4. Assessment of the Transcondylar Screw and Epicondylar Pin Accuracy

After completion of implant placement in all bone models, the accuracy of the transcondylar screw and epicondylar pin insertion was evaluated. The accuracy was determined by measuring the angular deviations of the transcondylar screws and epicondylar pins relative to their pre-planned trajectories, as well as by assessing the displacement of the screw exit point at the medial epicondyle compared to the planned exit location. By assessing the parameters, radiographs (X-rays) were used to measure the angle. A custom template was used to ensure all models were in the same position for angle measurements.

**Figure 4:**
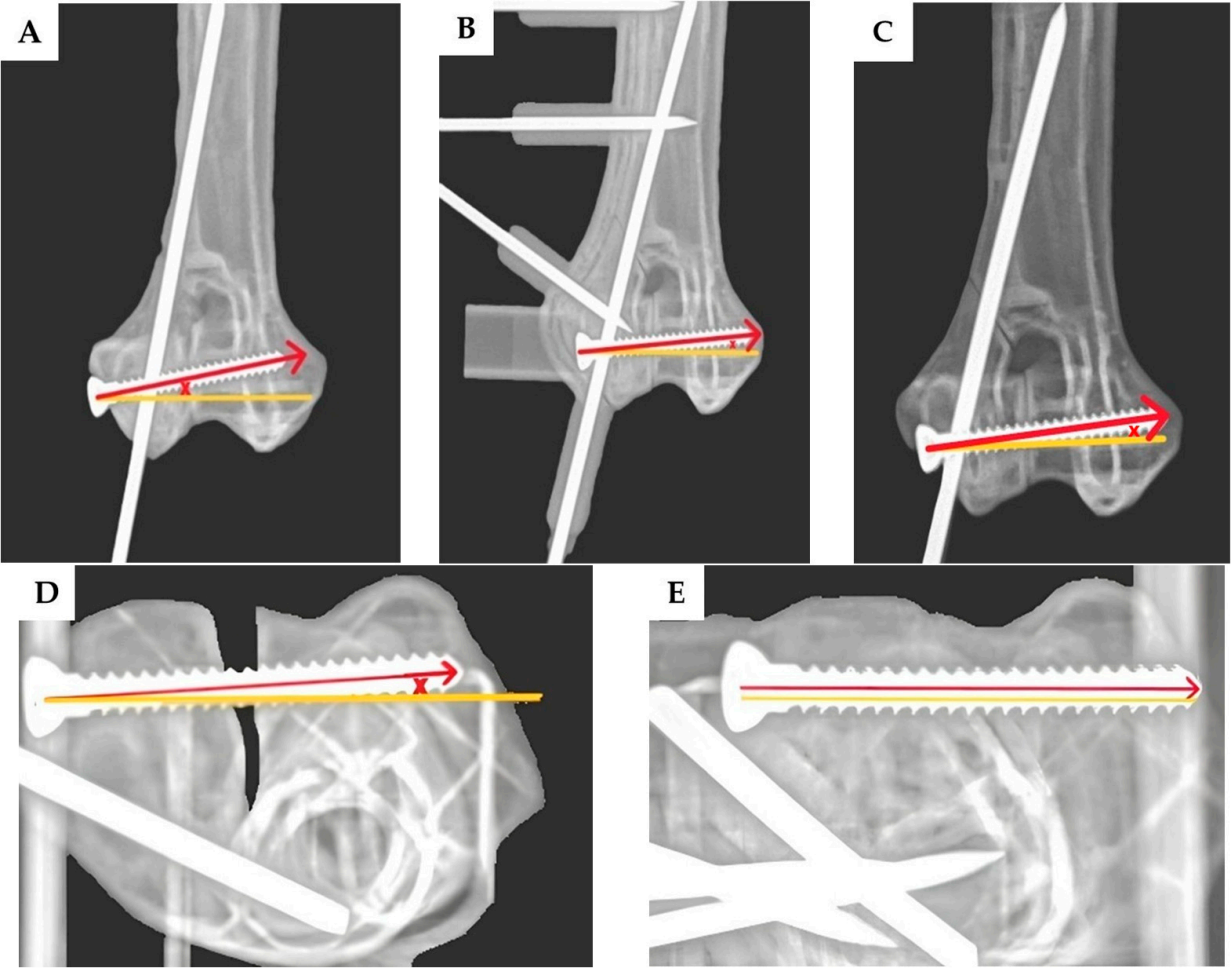
After inserting the transcondylar screw and epicondylar pin, the model was X-rayed to assess the transcondylar screw angle deviation, including both the proximodistal directional angle deviation and the craniocaudal directional angle deviation. The craniocaudal view radiographs (**A**–**C**) were used to assess the proximodistal directional angle deviation of the transcondylar screw, and the dorsoventral view radiographs (**D**,**E**) were used to assess the craniocaudal directional angle deviation of the transcondylar screw. The red arrow represents the direction of the actual transcondylar screw, while the yellow line represents the direction of the pre-planned transcondylar screw trajectory. The angle between the red arrow and the yellow line, marked by the symbol “×”, indicates the angle deviation of the transcondylar screw. The transcondylar screw angle deviation from both measurements was compared between the group without the 3D-printed drill guide (**A**,**D**) and the group with the 3D-printed drill guide (**B**,**E**). After the transcondylar screw and epicondylar pin were placed, the 3D-printed drill guide was removed (**C**). When the implant was placed without using a guide, an intercondylar fracture gap was sometimes observed, which was measured to be 2.5 mm according to the X-ray image (**D**).

**Figure 5:**
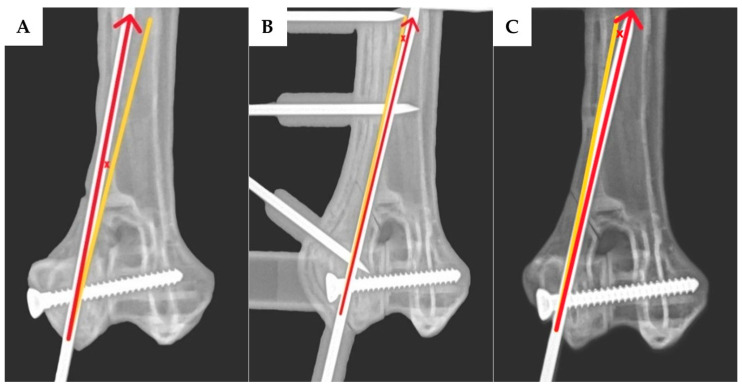

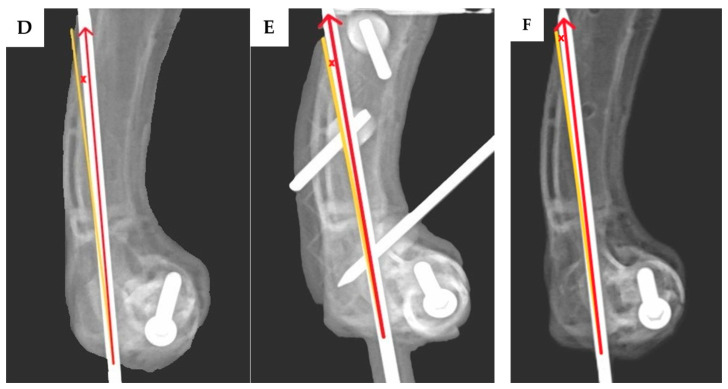
The model was X-rayed to assess the angle deviation of the epicondylar pin, including both the mediolateral directional angle deviation and the craniocaudal directional angle deviation. Craniocaudal view radiographs (**A**–**C**) were used to assess mediolateral directional angle deviation of the epicondylar pin, and lateral view radiographs (**D**–**F**) were used to assess the craniocaudal directional angle deviation of the epicondylar pin. The red arrow represents the direction of the actual epicondylar pin, while the yellow line represents the direction of the pre-planned epicondylar pin trajectory. The angle between the red arrow and the yellow line, marked by the symbol “×”, indicates the angle deviation of the epicondylar pin. The angle deviation of the epicondylar pin from both measurements was compared between the group not using a 3D-printed drill guide (**A**,**D**) and the group using a 3D-printed drill guide (**B**,**D**). After the transcondylar screw and epicondylar pin were placed, the 3D-printed drill guide was removed (**C**,**F**).

The angular deviation of the transcondylar screw was measured in two planes: the proximodistal direction was assessed using craniocaudal view radiographs (Figure 4A–C), while the craniocaudal direction was evaluated using dorsoventral view radiographs (Figure 4D,E). For the epicondylar pin, the angular deviations were measured in the mediolateral direction using craniocaudal view radiographs (Figure 5A–C) and in the craniocaudal direction using lateral view radiographs (Figure 5D–F). The exit point translation for the transcondylar screw was defined as the linear distance between the actual screw tip and the planned exit point at the medial epicondyle, as measured on the lateral view radiographs.

### 2.5. Statistical Analysis

This study was a comparison of experimental research, with a total of 60 bone model samples, divided into two groups of 30 samples each. Comparisons between the groups were conducted based on the transcondylar screw angle deviations, epicondylar pin angle deviations, and transcondylar screw exit point translation. These were treated as continuous variables. As the data distribution was determined to be non-parametric, statistical analysis was performed using the Mann–Whitney U test to compare the median values between the two groups. A *p*-value of less than 0.05 was considered statistically significant.

## 3. Results

**Figure 6:**
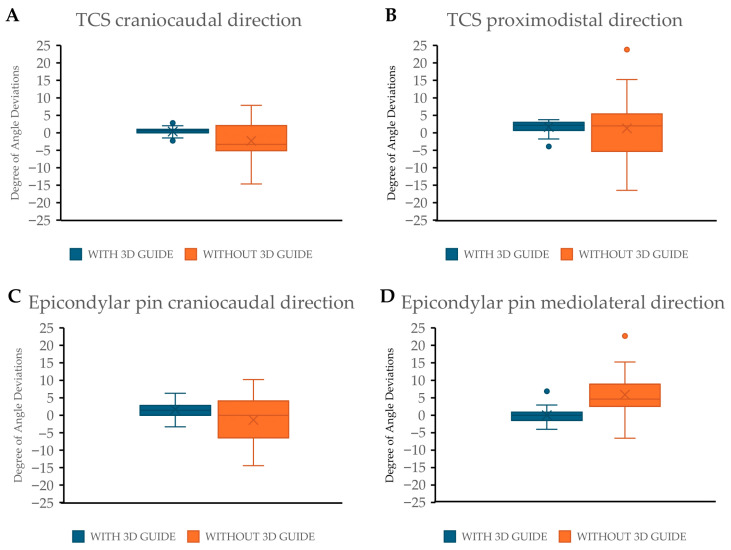
Once all the data were collected, statistical analysis was conducted. The results are presented as Box-and-Whisker plots. The data displayed include the angle deviation of the transcondylar screw (TCS) in the craniocaudal direction (**A**) and proximodistal direction (**B**), as well as the angle deviation of the epicondylar pin in the craniocaudal direction (**C**) and mediolateral direction (**D**). In the graphs, blue represents the group with the 3D-printed drill guide, and orange represents the group without the 3D-printed drill guide.

Following the collection of radiographic data and subsequent measurements of the transcondylar screw angle deviation, epicondylar pin angle deviation, and transcondylar screw exit point translation, statistical analyses were performed. The results demonstrated that the transcondylar screw angle deviation in the craniocaudal direction showed a statistically significant difference (*p* < 0.001) between the groups. The group with the 3D-printed drill guide had a median (range) deviation of 0.61° (−2.29–2.82°), whereas the group without the drill guide had a median (range) deviation of −3.29° (−14.63–7.85°), with the negative value indicating a tendency for the screw to deviate caudally (Figure 6A).

**Figure 7:**
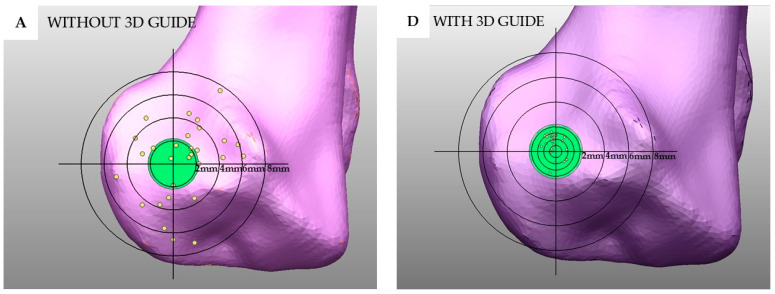

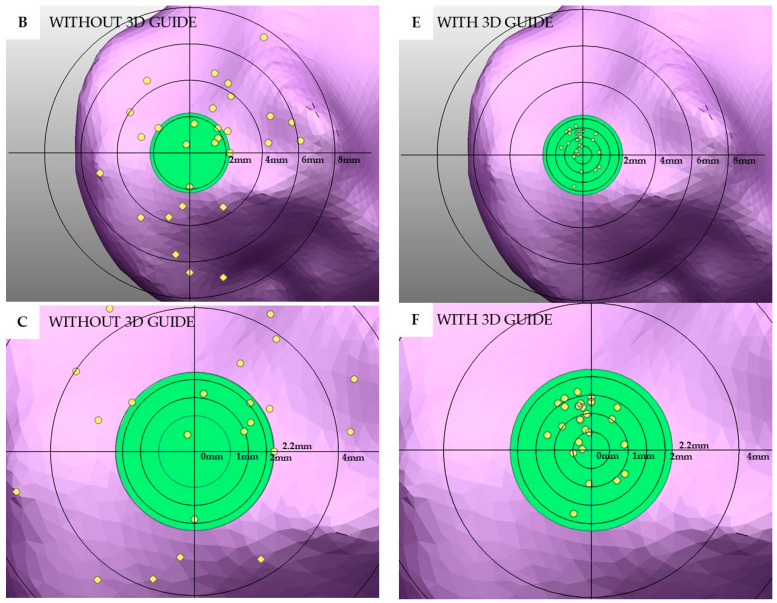
After measuring the transcondylar screw angle deviation and the exit point translation of the transcondylar screw, the exit point was plotted as positions in Computer-Aided Design (CAD) software. The exit point translation of the transcondylar screw for the group without the 3D-printed drill guide (**A**–**C**) and the exit point translation of the transcondylar screw for the group with the 3D-printed drill guide (**D**–**F**) are plotted on a graph overlaying the positions of the humeral bone. The center point of the circle represents the pre-planned position of the exit point of the transcondylar screw. The circles in the regions farther from the center have increased by 2 mm each, with the green circle having a radius of 2.2 mm. The yellow circle represents the exit point of the transcondylar screw. The group with the drill guide remains entirely within the green circle and shows less dispersion compared to the group without the drill guide.

For the transcondylar screw angle deviation in the proximodistal direction, a statistically significant difference was also observed (*p* < 0.001). The group with the drill guide showed a median of 2.13° (−3.9–3.76°) compared to 1.99° (−16.47–23.82°) in the no drill guide group (Figure 6B). In terms of the transcondylar screw exit point translation, the group with the drill guide had a median distance of 1.15 mm, significantly lower than the 3.80 mm observed in the no guide group (*p* < 0.001). The distribution of the exit point translation was also demonstrated using a plot graph (Figure 7).

Regarding the epicondylar pin, the angle deviation in the craniocaudal direction also showed a statistically significant difference (*p* < 0.001). The group with the drill guide showed a median (range) deviation of 1.42° (−3.31–6.29°), compared to 0.86° (−14.44–10.22°) in the no drill guide group (Figure 6C). Lastly, the epicondylar pin angle deviation in the mediolateral direction revealed a significant difference (*p* < 0.001), with the group with the drill guide demonstrating a median (range) deviation of 0° (−4.02–6.87°), compared to 4.63° (−6.57–22.69°) in the no drill guide group (Figure 6D). In the group with no drill guide, the mean (SD) fracture gap was found to be 1.02 (0.98) mm. In contrast, in the group with the drill guide, no fracture gap was observed.

## 4. Discussion

In our study, a French Bulldog was used as the model, since it has a relatively small humerus size compared to its body weight, and its bone structure is asymmetrical compared to other dog breeds. Previous studies have shown that humeral condylar fractures most commonly occur as lateral humeral condyle fractures [21,22,23]. This is primarily due to the force’s transmission from the radius to the humeral capitulum, which is then transferred to the lateral supracondylar crest. As the lateral side is structurally weaker than the medial side, the impact forces transmitted through the radius often result in fractures of the lateral humeral condyle [9]. In chondrodystrophic breeds, abnormal development of the distal ulnar physis can lead to humeroulnar subluxation and abnormal limb angulation, resulting in greater force transmission from the radial head to the humeral capitulum than in other breeds. Additionally, the humeral capitulum in these breeds tends to be eccentrically shaped, further increasing their susceptibility to lateral humeral condyle fractures [1,12]. Investigations since 2020 have highlighted an increasing incidence of humeral condylar fractures in both Spaniels and French Bulldogs. Spaniel breeds are more at risk for incomplete ossification centers compared to other breeds, which leads to a higher incidence of humeral condylar fractures [4,24,25]. Hence, we chose to study lateral humeral condylar fracture in French Bulldogs.

Among the various surgical techniques available for the repair of humeral condylar fractures, transcondylar screw fixation combined with epicondylar pinning is the most widely used method. The placement of a transcondylar screw in a lag fashion promotes direct cortical contact, which facilitates primary bone healing and reduces the risk of developing degenerative joint disease [6,26]. The epicondylar pin serves to counteract the rotational forces [27]. Nevertheless, previous studies have reported a high complication rate of 30–45% associated with the surgical repair of humeral condylar fractures, including implant failures such as screw loosening, screw breakage, pin migration, seroma formation, and surgical site infections [26,28]. One of the primary factors associated with these complications is the presence of an intercondylar fracture gap, which can be detected radiographically postoperatively. The gap is significantly related to screw breakage and to developing degenerative joint disease [26]. The angle deviation of the transcondylar screw is a critical factor: a previous study showed that for every 10° deviation from the ideal screw angle, the risk of developing an intercondylar fracture gap increases 4.82 times. Additionally, surgical durations exceeding 30 min increase the complication rate 2 times [26]. In our study, we recognized the importance of these complications and therefore sought to develop a method to enhance the surgical precision to reduce the likelihood of postoperative complications, using 3D technology.

Three-dimensional printing technology has been used for complicated surgical planning. Three-dimensional models generated from radiographic imaging, such as CT or MRI scans, allow clinicians to visualize complex anatomical structures, improve surgical training, reduce iatrogenic trauma, and decrease operative times [29,30,31]. In orthopedic surgery, 3D printing is commonly used to create patient-specific implants [32], pre-contoured plates, and surgical guides such as cutting guides [33], reaming guides, and drill guides to enhance the surgical precision. According to our study, in the group without the 3D-printed drill guide, there were some cases of an intercondylar fracture gap, as seen in Figure 3B and Figure 4D. The transcondylar fracture gap results from an excessive transcondylar screw angle. Based on the findings from this study [26], a transcondylar screw angle of 10 degrees allows for the calculation of the exit point translation of the transcondylar screw to remain within 10 degrees, which results in a translation of 2.2 mm from the center point. This is calculated by taking the cosine of 10 degrees of the distance from the medial epicondyle to the lateral epicondyle of the bone, which is 2.3 mm, resulting in a value of 2.2 mm. The green circle is defined as having a radius of 2.2 mm. In Figure 7D–F, the exit point of the transcondylar screw in the group with the 3D-printed drill guide is entirely within the green circle. While our experiment found that the placement of the transcondylar screw was not 100% identical to the planned position, the deviation was minimal—only a few millimeters—and it did not affect the transcondylar screw angle. Importantly, this small difference did not lead to the formation of an articular step or articular gap. Without the drill guide, an intercondylar fracture gap occurred. However, no intercondylar fracture gap was observed in the group with the drill guide. Hence, the use of the 3D-printed drill guide, as designed in this study, can reduce the risk of complications from implant failure and degenerative joint disease. Surgical time is another important parameter, as longer surgical durations are associated with a higher risk of complications [26]. In the cited study, the surgical time was measured from the start of the surgical approach to implant placement and concluded with skin closure. As our study was conducted on a bone model, we were unable to measure the time in the same way; so, data on the surgical time are not available. However, it is expected that the 3D-printed drill guide will help reduce the time spent in certain stages of the surgery, such as bone reduction and implant placement, ultimately shortening the overall surgical time.

In a study by Easter et al., 2020, using 3D patient-specific drill guides to address humeral intracondylar fissures, they demonstrated that placing transcondylar screws from the medial to lateral direction resulted in mean (SD) craniocaudal and proximodistal angular deviations of −2.91° (2.54°) and −3.00° (2.83°), respectively. In this study, we used a lateral-to-medial screw insertion technique and lag compression for the treatment of lateral humeral condyle fractures [15]. Furthermore, Barnes et al., 2014 investigated the safe corridors and tolerance angles for transcondylar screw placement and reported that the lateral-to-medial screw insertion provides a wider tolerance angle in the proximodistal direction than medial-to-lateral placement due to the anatomical structure of the epicondyles [34]. This supports the preference for lateral-to-medial insertion in minimizing complications. Our findings demonstrated that the use of the 3D-printed drill guide significantly improved the screw placement accuracy. The group with the drill guide exhibited craniocaudal and proximodistal angle deviations lower than the no drill guide group, with mean (SD) values of 0.37° (1.24°) and 1.65° (1.80°), respectively. These values were even lower than previous studies employing 3D-printed guides. Additionally, the exit point translation of the screw was notably smaller in the guide group compared to the no guide group. Similarly, the epicondylar pin showed lower angular deviations in both the craniocaudal and mediolateral directions when the drill guide was used. In this experiment, the transcondylar screws were inserted multiple times; hence, there might be a learning effect that allows for more accurate screw placement in subsequent attempts compared to the first. However, using a 3D-printed drill guide for transcondylar screw insertion helps to increase the accuracy the first time, even without prior experience.

In this study, we used plastic bone models to assess the accuracy and precision of the designed drill guide. Because the bone models were of uniform size, they were suitable for measuring the accuracy of the drill guide. On the other hand, if cadavers were used for study, the bones would have different characteristics, and it would not be possible to control the fractures to the same area. A 3D-printed bone model can have various materials and printing methods. We used the fused deposition modeling (FDM) 3D printer [35] and polylactic acid (PLA) as the material, because it is easy to operate and cost-effective. The study by Marturello et al. (2023) [36] compared the size of the humerus bone with different types of 3D printers, determining that the condylar region of the humerus closely resembled the original in size. Therefore, it is recommended that printed models be tested and verified before being used in actual surgical procedures. The PLA material was also used for the drill guide. However, PLA is sensitive to heat and melting points at 120 °C; hence, it must be sterilized via ionizing radiation and not via an autoclave. Other materials for orthopedic surgical guide include photopolymer resins, Acrylonitrile Butadiene Styrene (ABS), and Polyethylene Terephthalate Glycol-modified (PETG). Photopolymer resins have smooth surfaces, high resolution, and high precision and can be sterilized using an autoclave; however, they are more expensive than PLA. ABS is stronger and more heat-resistant than PLA and PETG; however, the printing process and post-processing steps are more difficult and complex. PETG is a developed material with properties in between PLA and ABS, offering better heat resistance and strength than PLA and being easier to print than ABS. It is a good option for use as a surgical guide.

Our 3D-printed drill guide was designed to allow easy switching between different drill bit sizes for both the thread hole and the gliding hole. This helps create a lag screw effect and makes the surgical procedure more convenient. The drill sleeves can be changed simply by removing the gliding hole and inserting the thread hole, without taking the guide off the humerus after it has been fixed in place. This is a major advantage, as it helps reduce the chance of errors caused by the misalignment of the drill bit with the original position. Our 3D-printed drill guide was designed based on the anatomical characteristics of the humerus; hence, the proximal part of the guide may come close to the radial nerve. However, the radial nerve can be retracted cranially to prevent nerve trauma from the 3D guide. Surgical management of humeral condylar fractures in French Bulldogs is particularly challenging, due to the greater degree of bone deformation compared to other breeds. The use of a transcondylar screw and epicondylar pin, guided by our custom-designed surgical guide, offers a promising alternative to enhance the surgical accuracy and efficiency. The time required to create the 3D-printed drill guide, starting from the DICOM files, begins with converting the files into STL format for the fractured bone and removing the surrounding soft tissue, which takes approximately 1 h. Next, the 3D-printed drill guide is designed to fit the specific bone, taking 12–24 h. The 3D printing process, including post-processing techniques, takes another 12 h. The final step is sterilization of the 3D-printed drill guide, which takes about 3 h. In total, from obtaining the CT scan images to having a ready-to-use 3D-printed drill guide, the process takes around 2–3 days. This time frame is not excessively long and is suitable for preparing the animal for surgery. Prior to clinical application, however, cadaveric testing is warranted to determine whether the outcomes remain consistent with those obtained in the plastic model. Confirmation of such findings would support subsequent implementation in live animal surgeries.

## 5. Conclusions

The experimental results show that the transcondylar screw angle deviation in both directions and the angle deviation of the epicondylar pin in both directions in the group with the designed 3D-printed drill guide were lower than those in the group without the guide. The 3D-printed drill guide has a good design, providing good therapeutic results by increasing the accuracy of the surgery, being easy to use, and having a short production time. It can serve as a model for designing treatments for lateral humeral condyle fractures in dogs of different ages or breeds, ensuring surgical precision and reducing the risk of postoperative complications.

## Data Availability

The original contributions presented in this study are included in the article. Further inquiries can be directed to the corresponding author.
